# Starting Dialysis on Time, At Home on the Right Therapy (START): Description of an Intervention to Increase the Safe and Effective Use of Peritoneal Dialysis

**DOI:** 10.1177/20543581211003764

**Published:** 2021-03-31

**Authors:** Robert R. Quinn, Farah Mohamed, Robert Pauly, Tracy Schwartz, Nairne Scott-Douglas, Louise Morrin, Anita Kozinski, Braden J. Manns, Scott Klarenbach, Alix Clarke, Danielle E. Fox, Matthew J. Oliver

**Affiliations:** 1 Cumming School of Medicine, University of Calgary, AB, Canada; 2 Department of Community Health Sciences, University of Calgary, AB, Canada; 3 Alberta Health Services, Edmonton, Canada; 4 Department of Medicine, University of Alberta, Edmonton, Canada; 5 Institute of Health Economics, Edmonton, Canada; 6 Department of Medicine, Division of Nephrology, University of Toronto, ON, Canada

**Keywords:** end-stage kidney disease, peritoneal dialysis, quality improvement, audit-and-feedback, protocol

## Abstract

**Background:**

Most of the patients with end-stage kidney failure are treated with dialysis. Jurisdictions around the world are actively promoting peritoneal dialysis (PD) because it is equivalent to hemodialysis in terms of clinical outcomes, but is less costly. Unfortunately, PD penetration remains low.

**Objectives:**

The Starting dialysis on Time, At Home, on the Right Therapy (START) Project had 2 overarching goals: (1) to provide information that would help programs increase the safe and effective use of PD, and (2) to reduce inappropriate, early initiation of dialysis in patients with kidney failure. In this article, we focus on the first objective and describe the rationale for START and the methods employed.

**Design:**

The START Project was a comprehensive, province-wide quality improvement intervention.

**Setting:**

The START project was implemented in both Alberta Kidney Care (AKC)-South and AKC-North, including all 7 renal programs in the province.

**Patients:**

The project included all patients who commenced maintenance dialysis between October 1, 2015, and March 31, 2018, in Alberta, Canada who met our inclusion criteria.

**Measurements:**

We reported baseline characteristics of incident dialysis patients overall, and by site. Our key performance indicator was the proportion of patients who received PD for any period of time within 180 days of the first dialysis treatment. Reports also included detailed metrics pertaining to the 6 steps in the process of modality selection and we had the capacity to provide more granular data on an as-needed basis. To understand loss of PD patients, we reported the numbers of incident patients who recovered kidney function, experienced technique failure, received a transplant, were lost to follow-up, transferred to another program, or died.

**Methods:**

START provided dialysis programs with a conceptual framework for understanding the drivers of PD utilization. High-quality, detailed data were collected using a tool that was custom-built for this purpose, and were mapped to steps in the process of care that drove the outcomes of interest. This allowed sites to identify gaps in care, develop action plans, and implement local interventions to address them. The process was supported by an Innovation Learning Collaborative consisting of 3 learning sessions that brought frontline staff together from across the province to share strategies and learnings. Ongoing data collection allowed teams to determine whether their interventions were effective at each subsequent learning session, and to revisit their interventions if required (the “Plan-Do-Study-Act Cycle”).

**Results:**

Future work will report on the impact of the START project on incident PD utilization at a provincial and regional level.

**Limitations:**

The time required to design and implement interventions in practice, as well as the need for multiple PDSA (Plan-Do-Study-Act) cycles to see results, meant that the true potential may not be realized during a relatively short intervention period. Change required buy-in and support from local and provincial leadership and frontline staff. In the absence of accountability for local performance, we relied on the goodwill of participating programs to use the information and resources provided to effect change. Finally, the burden of documentation and data collection for frontline staff was high at baseline. We anticipated that adding supplemental data collection would be difficult.

**Conclusions:**

The START project was a comprehensive, province-wide initiative to maximize the safe and effective use of PD in Alberta, Canada. It standardized the management of incident dialysis patients, leveraged high-quality data to facilitate the reporting of metrics mapped to steps in the process of care that drove incident PD utilization, and helped programs to identify gaps in care and target them for improvement. Future work will report on the impact of the program on incident utilization at the provincial and regional level.

## Abrégé

**Contexte:**

La majorité des patients atteints d’insuffisance rénale terminale (IRT) sont traités par dialyze. On s’efforce, partout dans le monde, de promouvoir la dialyze péritonéale (DP) parce qu’elle donne des résultats cliniques équivalents à ceux de l’hémodialyse, mais à moindres coûts. Malheureusement, l’adhésion à la DP demeure faible.

**Objectifs:**

Le projet START (*The Starting dialysis on Time, At Home, on the Right Therapy*) avait deux objectifs principaux : (1) fournir de l’information qui aiderait les programs à accroître l’utilization sûre et efficace de la DP, et (2) réduire l’initiation précoce et inappropriée de la dialyze chez les patients atteints d’insuffisance rénale. Dans cet article, nous nous concentrons sur le premier objectif et nous décrivons la raison d’être de START et les méthodes employées.

**Type d’étude:**

Le projet START était une intervention globale d’amélioration de la qualité à l’échelle provinciale.

**Cadre:**

Le projet START a été mis en œuvre au sein de l’Alberta Kidney Care (AKC) — South et de l’AKC-North, qui incluent les 7 programs rénaux de la province.

**Sujets:**

Le projet a inclus tous les patients répondant aux critères d’inclusion qui avaient amorcé des traitements de dialyze d’entretien entre le 1er octobre 2015 et le 31 mars 2018 en Alberta (Canada).

**Mesures:**

Nous avons rapporté les caractéristiques de base des patients dialysés incidents de façon globale et par site. Notre principal indicateur de performance était la proportion de patients qui ont reçu la DP pendant une période quelconque au cours des 180 jours suivant le premier traitement de dialyze. Les rapports comprenaient également des mesures détaillées concernant les 6 étapes du processus de sélection de la modalité et nous étions en mesure de fournir des données plus précises au besoin. Pour mieux définir la perte de patients sur DP, nous avons rapporté le nombre de patients incidents ayant récupéré une fonction rénale, ayant vécu une défaillance technique, ayant reçu une greffe, ayant été perdus de vue, ayant été transférés à un autre program ou étant décédés.

**Méthodologie:**

START a fourni aux programs de dialyze un cadre conceptuel pour comprendre les facteurs d’utilization de la DP. Des données détaillées et de grande qualité ont été recueillies à l’aide d’un outil conçu spécifiquement à cette fin, et ont été mises en correspondance avec les étapes du processus de soins ayant conduit aux résultats d’intérêt. Ainsi, les sites ont pu déceler les lacunes dans les soins, élaborer des plans d’action et mettre en œuvre des interventions locales pour combler ces lacunes. Le processus a été appuyé par une collaboration d’apprentissage innovante constituée de trois séances d’apprentissage qui ont réuni le personnel de première ligne de toute la province afin que ceux-ci partagent leurs stratégies et apprentissages. La collecte continue de données a permis aux équipes de déterminer si leurs interventions étaient efficaces et de revoir leurs interventions si nécessaire (le cycle PDSA: *Plan-Do-Study-Act*) lors de chaque séance d’apprentissage ultérieure.

**Résultats:**

Les travaux futurs feront état de l’impact du projet START sur l’utilization de la DP à l’échelle provinciale et régionale.

**Limites:**

Le véritable potentiel du projet pourrait ne pas se révéler lors d’une période d’intervention relativement courte en raison du temps requis pour concevoir les interventions et les mettre en œuvre dans la pratique, et parce que le processus nécessite de procéder à plusieurs cycles PDSA avant de produire des résultats. Le changement exige l’adhésion et le soutien des directions locales et provinciales et du personnel de première ligne. En l’absence de responsabilisation à l’égard des réalisations locales, nous nous sommes appuyés sur la bonne volonté des programs participants à utiliser l’information et les ressources fournies pour faire le changement. Aussi, en début de processus, le fardeau de documenter et de collecter les données s’est avéré lourd pour le personnel de première ligne. Nous avions prévu qu’il serait difficile d’ajouter une collecte de données supplémentaire.

**Conclusion:**

Le projet START était une initiative globale à l’échelle provinciale qui visait à maximiser l’utilization sécuritaire et efficace de la DP en Alberta, au Canada. Le projet a normalisé la gestion des patients dialysés incidents, a exploité des données de haute qualité pour faciliter la déclaration des indicateurs correspondant aux étapes du processus de soins menant à l’utilization de la DP, et a aidé les programs à déceler les lacunes de soins et à les améliorer. Les travaux futurs feront état de l’impact du program sur l’utilization incidente au niveau régional et provincial.

## Introduction

Most patients with end-stage kidney failure are treated with dialysis. Although they represent 0.15% of the population, they consume up to 4% of health care expenditures in developed countries.^1^ Most patients with kidney failure are treated with hemodialysis (HD), but peritoneal dialysis (PD) is an equivalent therapy with respect to important clinical outcomes and costs between US$39 000 and US$57 000 less, per patient-year, to provide.^2-5^ Jurisdictions around the world are actively promoting PD, but with few exceptions, penetration remains low.^6-8^

Maximizing PD utilization among suitable patients is a complex problem and is influenced by a number of factors. To increase program utilization of PD, the use of the treatment in incident patients must be maximized, time on therapy must be optimized, and loss of patients to HD must be minimized. Focusing on incident PD utilization is a critical first step and for most of the jurisdictions that provide patients with a choice of dialysis therapy, this process requires that programs identify all patients initiated on or approaching the need for dialysis, educate them about their treatment options, determine if they are eligible, and offer them the therapy. Once offered, patients must choose PD, have a PD catheter placed, and then start on PD.^9^ Maximizing incident PD utilization means optimizing all of these steps.

A recent scoping review of strategies to increase PD utilization identified patient-, provider-, and policy-level interventions aimed at increasing PD utilization.^10^ Enhanced modality education programs, audit-and-feedback, providing assisted PD, and the introduction of bedside PD catheter placement were associated with increased PD utilization. However, these interventions targeted individual steps in the process of care and typically, were applied on a small scale. The implementation of a PD First Policy was associated with an increase in PD utilization, but involved restricting patient choice, and high-quality data about outcomes on therapy were lacking.^10^

We hypothesized that providing programs with comprehensive high-quality data, collected on a common platform, about all steps in the process of care that drive incident PD utilization, might enable them to identify problem areas, design and implement interventions to address them, and facilitate monitoring for improvement. This project was called the Starting dialysis on Time, At Home, on the Right Therapy (START) Project and had 2 overarching goals: (1) to provide information that would help programs increase the safe and effective use of PD, and (2) to reduce inappropriate, early initiation of dialysis in patients with kidney failure in Alberta, Canada. In this article, we focus on the first objective and describe the rationale for START and the methods employed.

## Methods

### Setting

Alberta Health Services (AHS) is a province-wide, integrated health system, responsible for delivering all specialty and hospital services to approximately 4.3 million people living in Canada’s fourth largest province. The START project was implemented in all Alberta Kidney Care (AKC)-South and AKC-North programs, including all 7 AHS chronic kidney disease (CKD) clinics: Calgary, Lethbridge, Medicine Hat, Edmonton (including University of Alberta Hospital [UAH] Gray Nuns Community Hospital [GNCH], and Royal Alexandra Hospital [RAH]), and Red Deer Regional Hospital Centre. The organization of care differed somewhat according to location. In smaller centers, like Lethbridge or Medicine Hat, advanced CKD care and dialysis were provided at the same location and team members often were cross-trained and participated in the care of different types of patients. In the Calgary Zone, advanced CKD care is based out of a single physical location and the nurses are dedicated to the care of CKD patients. PD and Home HD nurses are based out of the same center, but in-center HD care is distributed among a number of dialysis units and staffed by dedicated nursing staff. Edmonton is similar to Calgary, but there is a dedicated physician group for home dialysis. In Red Deer, advanced CKD and dialysis care are based out of a single location.

### Population

Our study included all patients who commenced dialysis in Alberta, Canada, during the “Pre-Intervention Period” (October 1, 2015, to September 30, 2016) and the “Intervention Period” (October 1, 2016, to March 31, 2018) and met one of the following inclusion criteria:

- End-stage kidney disease, in the opinion of their nephrologist, and initiated dialysis therapy;
- Acute kidney injury and required dialysis for ≥28 days;
- Received ≥1 outpatient dialysis treatment;
- Had a failed transplant and initiated dialysis therapy.

We excluded patients who transferred into a program from another center who were already established on dialysis therapy. Our intention was to isolate a population of patients that was faced with a modality choice and were appropriate for modality education.

### Data Sources

#### Establishing baseline measures (October 1, 2015, to September 30, 2016)

AKC-North and AKC-South provided historical data about incident PD utilization. These historical data were used to establish the baseline for the primary outcomes identified as part of the START Project. In AKC-South, information was obtained from a local dialysis database.^11^ In AKC-North, chart review was required as comprehensive data were not available in existing electronic systems. Reliable information regarding baseline patient characteristics and important process measures was not available prior to the implementation of the START project (October 1, 2016).

#### Reporting during the intervention period (October 1, 2016 to March 31, 2018)

Data were collected using the Dialysis Measurement Analysis and Reporting (DMAR™) System that has been used previously in several Canadian provinces and during a pilot project conducted in the Calgary Zone between 2013 and 2014.^2,9,12-20^ Baseline information about pre-dialysis care, the circumstances surrounding the initiation of dialysis (including the indication for starting and the presence or absence of symptoms and signs), baseline demographics, comorbidities, and laboratory information were recorded. We also captured a structured assessment for PD eligibility including contraindications to therapy, medical, social, and psychological barriers to home dialysis, patients’ living situations, details of the support available to them in their homes, whether they were ultimately deemed eligible for PD by the multidisciplinary team, whether they were offered PD as a treatment option, chose PD, and ultimately received it. Information regarding important clinical outcomes was captured including changes in treatment status (type of dialysis, level of assistance, location of dialysis), the occurrence of hospital admissions and interventional procedures, recovery of kidney function, transplantation, outmigration, and death.

### Intervention

The START project was a complex intervention that was rolled-out in 3 phases^21^:

#### Phase 1: We created the infrastructure to support collection of high-quality data tied to the 6-step process of modality selection and dialysis initiation

After completing a privacy impact assessment and finalizing data sharing agreements, the Dialysis Measurement Analysis and Reporting (DMAR™) System was installed in the AHS environment and tested at all participating sites, province-wide. Multidisciplinary stakeholders at all sites were trained to organize and lead multidisciplinary meetings where all new dialysis patients were identified and tracked on a roster. They ensured all new dialysis starts in their programs were identified, assessed to determine their eligibility for PD, educated about their treatment options, offered PD if they were eligible, supported to make an informed modality decision, and initiated on their chosen therapy. Patients remained on the meeting roster and were discussed at each multidisciplinary meeting until they had completed the process described above. Barriers to PD were identified and discussed and potential solutions were brainstormed. In addition, gaps in provider knowledge were identified providing opportunities for targeted education. The composition of the multidisciplinary team was variable depending on the site, but included physicians, home dialysis nurses, nurses from the kidney clinics, managers, and social workers. We encouraged sites to meet every 2 weeks, but frequency of the meetings was at the discretion of the programs. Staff were trained to document this process and the relevant variables in a standardized manner in the data system, ensuring that all sites collected data on a common platform, with a consistent coding schema to permit objective comparisons across sites and programs. To complement data collection efforts, all sites also implemented a standardized process to guide patients through the process of modality selection.

#### Phase 2: Structured review of all new dialysis patients and reporting of metrics tied to the process of modality selection and dialysis initiation

Data capture began on October 1, 2016. All data were reviewed centrally for consistency in coding, accuracy, and completeness by expert reviewers (R.R.Q. and F.M.). This process was particularly important for areas that are inherently subjective, such as the assessment of PD eligibility. Queries were communicated back to the user who had entered the data and were addressed prior to analysis and reporting, ensuring a high-quality data set.

**Figure 1.**
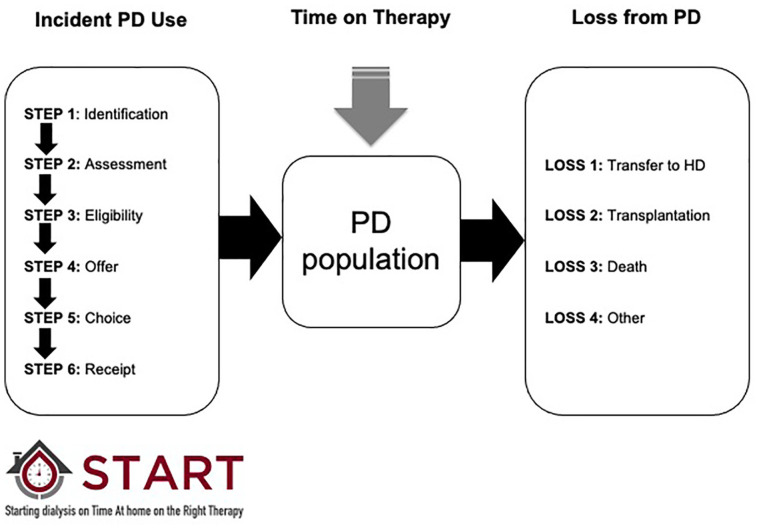
A conceptual framework for understanding the drivers of PD utilization at the program level. The framework highlights 6 steps that drive incident PD use (identification, assessment, determination of eligibility, offer of PD, patient choice, and receipt of PD) and 4 main causes of loss (transfer to HD, transplantation, death, and other including transfer to another program and recovery of kidney function). Independent of the number of patients who start PD and the number of patients lost, time on therapy is an important contributor to overall PD prevalence. The framework guided data collection efforts, reporting, and the development of local action plans. PD = peritoneal dialysis; HD = hemodialysis.

**Figure 2.**
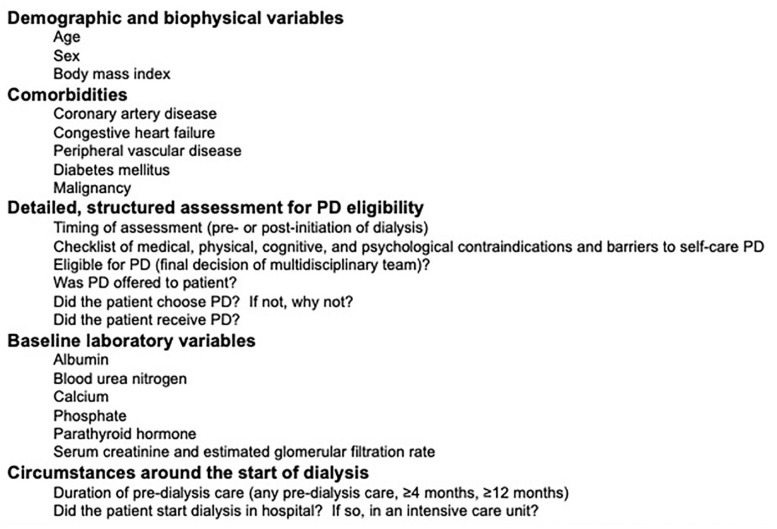
Baseline data captured on all patients enrolled in the START project included demographic and biophysical variables, comorbidities, a detailed assessment for PD eligibility, baseline laboratory variables, and details of the circumstances surrounding the start of dialysis. PD = peritoneal dialysis.

A conceptual framework for understanding the drivers of PD utilization is shown in Figure 1. Data collection and reporting for START were built around this framework. We reported baseline characteristics of incident dialysis patients overall, and by site, including age, demographics, body mass index, comorbidities, a detailed PD assessment, and baseline laboratory variables. We also reported the proportion of patients who received pre-dialysis care, started dialysis in hospital, and started dialysis in an intensive care unit. Details are included in Figure 2.

**Figure 3.**
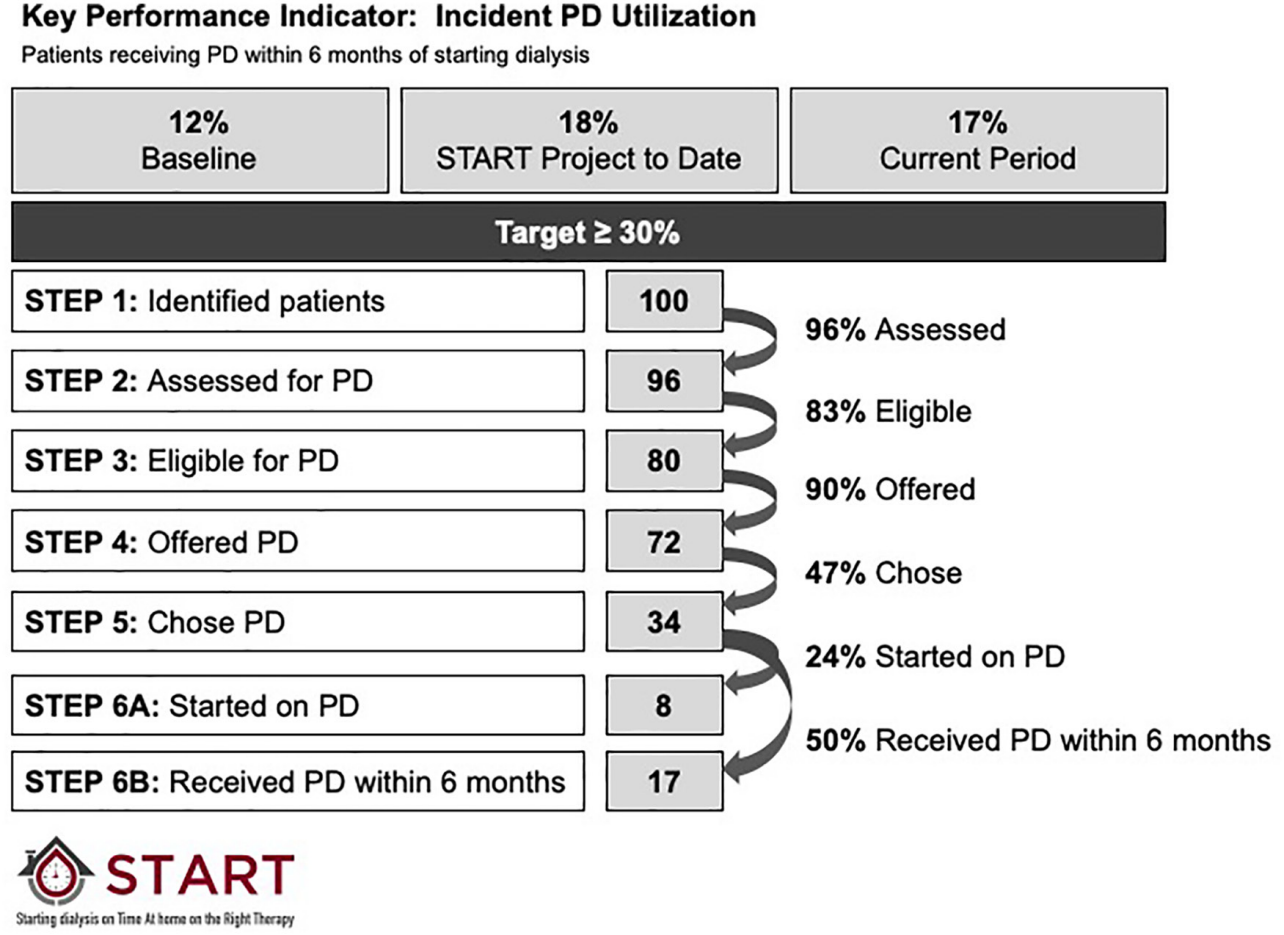
Sample report for the Key Performance Indicator: Incident PD Utilization. Baseline performance, cumulative performance to date during the START project, and data from the current period were reported. Target incident PD utilization was greater than or equal to 30%, based on stakeholder consensus that a 5% increase from baseline would be the provincial goal. Below this, detailed information about the number and percentage of patients making it through each step in the process of care was provided to each site. “Started on PD” meant that the patient received PD as the very first treatment modality. “Received PD within 6 months” meant that the patient received PD at some point during the first 6 months of dialysis therapy. PD = peritoneal dialysis.

The primary goal of the START Project was to improve the use of incident PD. To measure progress, we reported our Key Performance Indicator, Incident PD use. This was calculated as the proportion of patients who received PD for any period of time within 180 days of the first dialysis treatment. This allowed time for patients who started dialysis urgently on hemodialysis to be converted to PD, if that was their intended choice. Reports included the baseline value (historical period), target value, and current value for the reporting period of interest. Results were presented overall, and by site (Figure 3).

**Figure 4.**
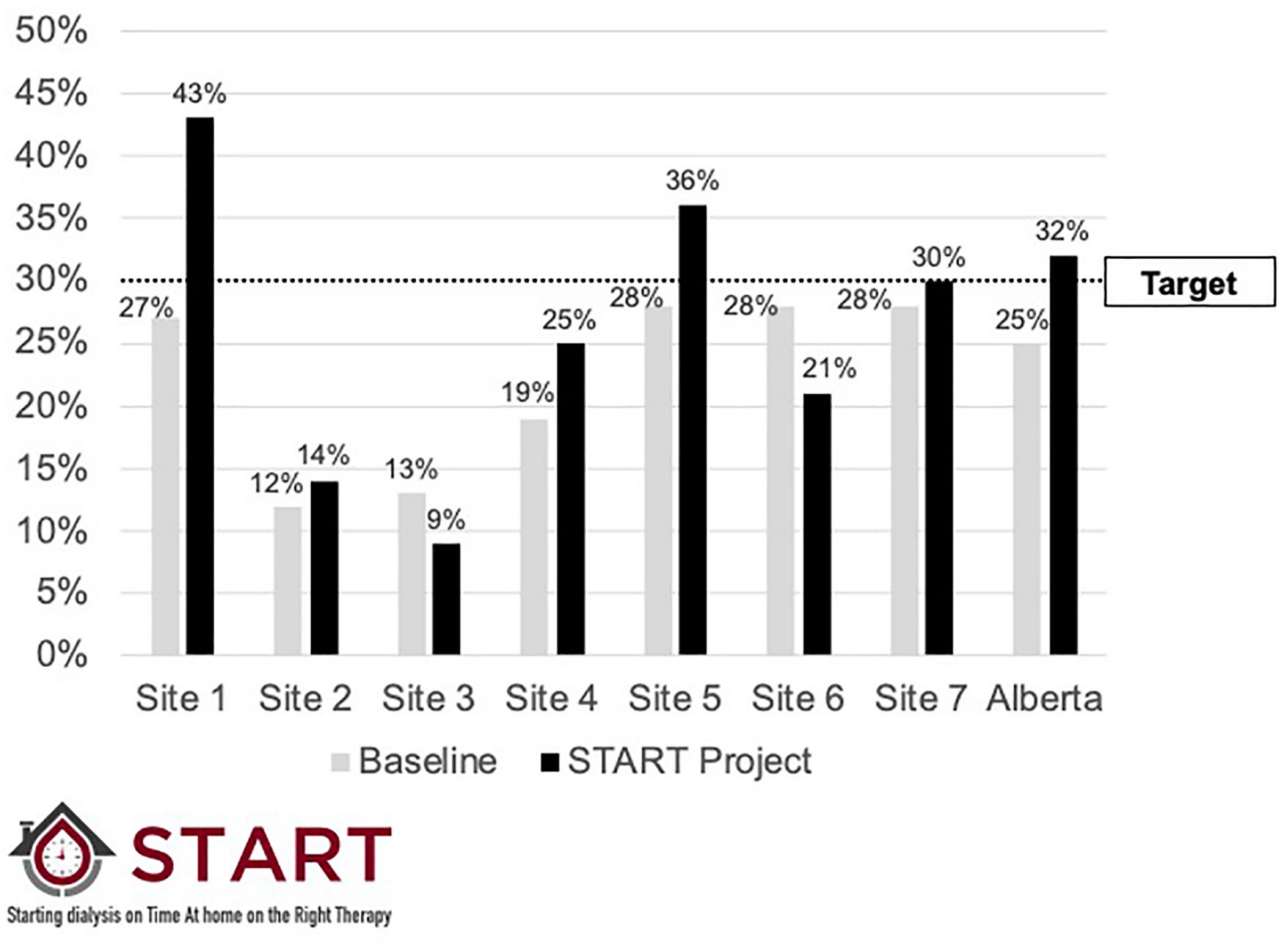
Sample report comparing incident PD utilization at baseline and cumulative performance to date during the START project for participating sites individually, and for the entire province of Alberta. PD = peritoneal dialysis.

**Figure 5.**
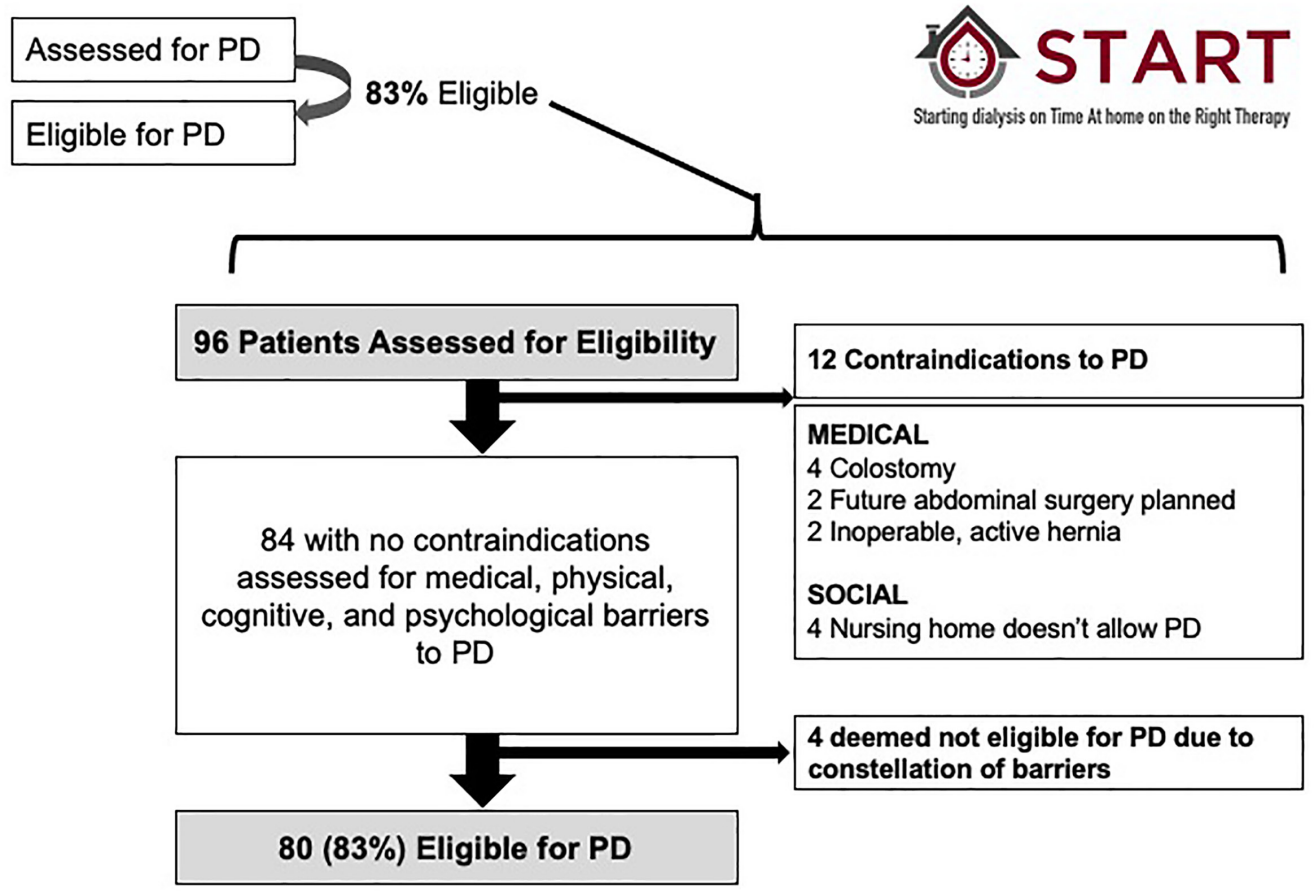
Sample report containing detailed information regarding decisions about PD eligibility in a participating program. In this case, 83% of patients assessed for PD were deemed eligible. Of the 96 patients assessed, 12 had a medical or social contraindication to PD. The details of the individual reasons are reported. Of the remaining 84 patients, an additional 4 patients had barriers that the multidisciplinary team felt could not be overcome and were also deemed ineligible for PD. This meant that 80 of the 96 patients (83%) assessed were eligible for PD. Note that the local multidisciplinary teams determined what constituted contraindications and barriers to therapy. Review of the individual reasons often identified gaps in provider knowledge and opportunities for education. PD = peritoneal dialysis.

Reports also included detailed metrics pertaining to the 6 steps in the process of modality selection during the Intervention Period. To understand drivers of incident PD utilization in a local program, we reported the percentage of patients who made it through the 6 steps required to start a patient on PD and benchmarked performance across sites in Alberta.^9^ Specifically, we reported the number of patients making it through each step and the percentage of patients moving from one step to the next overall, and by site (Figures 3 and 4). We had the capacity to perform a more detailed analysis on any of these secondary outcomes to provide more granular data on an as-needed basis (an example is included as Figure 5).

To understand loss of PD patients, we reported the numbers of incident patients who recovered kidney function, experienced technique failure, received a transplant, and who died. We also reported other causes of loss including loss to follow-up and transfer to another program.

#### Phase 3: Support of quality improvement process using a modified innovation collaborative

Participating teams were provided with site-specific reports on a quarterly basis. In parallel with this, we implemented an Innovation Learning Collaborative modeled on The Institute for Healthcare Improvement’s (IHI) Collaborative Model for Achieving Breakthrough Improvement,^22^ consisting of 3 learning sessions (June 2017, December 2017, and March 2018). These sessions brought frontline staff together from across the province. Data were mapped to steps in the process of care that drove the outcomes of interest, allowing sites to identify local barriers and opportunities to improve. The teams developed action plans and implemented local interventions to address these barriers. Ongoing data collection allowed teams to determine whether their interventions were effective at each subsequent learning session, and to revisit their interventions if required (the “Plan-Do-Study-Act Cycle”).^23^ The forum also provided sites with the opportunity to share local practices and learn from one another.

### Ethical Considerations

To comply with Alberta Health Services’ (AHS) privacy standards, we only reported program- and site-specific data as aggregate health information. If sample sizes were less than 10 patients, we suppressed them to minimize the risk of identifying individual patients. In these situations, relevant information was communicated directly to the sites, programs, and staff involved in the care of these patients, but not reported publicly.

## Discussion

START was a comprehensive, province-wide intervention introduced in Alberta, Canada, between October 1, 2016, and March 31, 2018. START provided dialysis programs with a conceptual framework for understanding the drivers of PD utilization. High-quality, detailed data were collected using a collection tool that was custom-built for this purpose, and were mapped to steps in the process of care that drove the outcomes of interest. This allowed sites to identify gaps in care, develop action plans, and implement local interventions to address them. The process was supported by an Innovation Learning Collaborative consisting of 3 learning sessions that brought frontline staff together from across the province to share strategies and learnings. Ongoing data collection allowed teams to determine whether their interventions were effective at each subsequent learning session, and to revisit their interventions if required (the “Plan-Do-Study-Act Cycle”).

Prior to START, we conducted a scoping review of strategies to increase PD utilization, modeled on the same conceptual framework.^9,10^ We identified patient-, provider-, and policy-level interventions aimed at increasing PD utilization. The overall quality of the evidence was low-to-moderate and the interventions tended to address a specific issue or problem and focused either on the outcome of PD use or an outcome that was a surrogate for increased PD utilization. Certain approaches, including the implementation of a PD First Policy, enhanced modality education programs, audit-and-feedback programs, assisted PD, and the introduction of bedside PD catheter placement were associated with increases in PD utilization. However, there were several important considerations. First, many studies lacked a control group and simply performed a before-and-after analysis. Although this approach has limitations, it is sometimes difficult to use more robust designs to evaluate quality improvement initiatives. Other studies used surrogates for increased PD utilization (eg, PD choice) that do not necessarily translate into increased PD uptake. Some interventions involved introducing infrastructure that did not previously exist, raising the question of whether or not interventions would be effective in more developed PD programs, and many studies were conducted in small centers or jurisdictions with very low PD penetration and had limited generalizability to programs with higher rates of PD use, or larger areas. Finally, very few reports included data to explain how increases in PD utilization were realized, making it difficult to transfer learnings to other programs.

Maximizing the safe and effective utilization of PD and improving the timing of dialysis initiation are complex problems with many moving parts. The approach taken in the START project was based on the belief that measuring current process of care comprehensively and understanding where there were local barriers was a necessary first step to solving problems efficiently. It also builds on the foundation of literature that suggests audit and feedback has been successful in improving outcomes in healthcare, particularly when based on reliable data, and when clear targets are provided along with an action plan that outlines the steps necessary to improve.^24^ Our prior experiences suggested that programs often have different challenges. Implementing interventions to improve care without first demonstrating there is a problem, leads to inefficient use of limited resources. For example, revamping modality education to increase choice rates is unlikely to be successful in a program where 50% to 60% of the people who are offered PD already choose it. In addition, we often are faced with small sample sizes in quality improvement and must answer the question of “did this intervention work?”. Measuring the process that drives an outcome of interest also allows programs to ensure that the interventions employed have impacted on the specific steps in the process of care *that were targeted*, and that subsequently translated into an impact on the key performance indicator of interest. When the focus is solely on high-level indicators, there may be an unforeseen or unmeasured change that influences the observed result. In those situations, one could come to the erroneous conclusion that an intervention was successful or unsuccessful, simply because process was not measured.

Our choice of Key Performance Indicators was deliberate. Although we tracked the proportion of patients who received PD as their first dialysis therapy, we knew from prior experience that it could take up to 180 days after the initiation of dialysis for some patients to receive their desired therapy.^9^ This was particularly true for patients who started urgently on HD, those who had limited or no pre-dialysis care, and in programs where there were long waits for PD catheter placement or PD training. As a result, our Key Performance Indicator for incident PD utilization was the receipt of PD within 180 days of dialysis initiation. The proportion of all starts receiving PD at any point during their treatment history was captured as a secondary outcome.

We anticipated several challenges with this project. First, simply cleaning up process and instituting data collection may change behavior in programs and lead to improvements in Key Performance Indicators, but we also hypothesized that programs would take time to address gaps in care that were identified through data collection and reporting. Given the short duration of the program, the time required to design and implement interventions in practice, as well as the frequent need for multiple PDSA cycles to fine tune interventions and see results, we anticipated that this work may not be completed by the end of the START project and the true potential may not be realized. Second, we anticipated that we would be able to efficiently identify areas for improvement in each participating program, but change would require buy-in and support from local and provincial leadership and frontline staff. Even with robust data and support for programs through learning collaboratives and expert consultation, in the absence of accountability for local performance, we relied on the goodwill of participating programs to use the information and resources provided to effect change. Finally, frontline staff are busy and the current burden of documentation and data collection is high. We anticipated that adding supplemental data collection would be difficult. We ensured the user interface was well-designed, objective and easy to understand, linked to definitions, and we provided training and mentoring to all data collection staff. We reviewed all data submitted and provided feedback directly to end-users. The staff responsible for data entry were intimately involved in the quality improvement process and saw the value of the information they helped to collect and how it was being used.

In summary, the START project was a comprehensive, province-wide initiative to maximize the safe and effective use of PD in Alberta, Canada. It standardized the management of incident dialysis patients, leveraged high-quality data to facilitate the reporting of metrics mapped to steps in the process of care that drove incident PD utilization, and helped programs to identify gaps in care and target them for improvement. Future work will report on the impact of the START project on incident PD utilization at a provincial and regional level.
